# Supplementary material for: Passenger and freight travel patterns: A cluster analysis based on urban networks
Source: PLoS One. 2025 Mar 11;20(3):e0318084. doi: 10.1371/journal.pone.0318084 (PMC11896054; doi:10.1371/journal.pone.0318084)
Supplement: Attachment — Submitted filename: Reponse_to_Reviewer4.docx [file pone.0318084.s001.docx]

**Dear Reviewer 4,**

Thank you for your detailed and insightful review of our manuscript. We appreciate the time and effort you have taken to provide us with constructive feedback. Below, we address each of the major comments you raised.

**Major Comment 1: The study only covers one year (2019), and it would be better to include longer periods of time.**

You suggested adding a separate section on the objectives of the study to improve the clarity and structure of the manuscript.

**Response:** Thank you very much for your valuable feedback. We fully agree that a longer time frame could provide additional insights into trends over time. However, the choice to use 2019 data was driven by the availability and quality of comprehensive traffic and freight flow data for that specific year. While analyzing multiple years could offer deeper insights, the 2019 data provides a strong and representative snapshot of regional traffic flows and economic activity. Furthermore, this study aims to establish a foundation for understanding key patterns in passenger and freight movement, which can be expanded in future research with longer time periods. We believe that, despite this limitation, the study's findings offer meaningful insights into regional connectivity and network analysis.

**Major Comment 2: Figures 3, 4, 5, and 6 do not include details and suggest modifying or deleting them.**

You suggested that Figures 3, 4, 5, and 6 lack sufficient details, and proposed modifying or removing them.

**Response:** Thank you for your constructive feedback. We have carefully reviewed the figures and agree that they could benefit from additional details. In response, we have revised Figures 3, 4, 5, and 6 by adding more detailed labels and descriptions to enhance clarity and comprehension. We believe these revisions will make the figures more informative and align better with the overall findings of the study. We have also included a more thorough explanation of each figure in the figure captions to ensure that their relevance to the analysis is clear.

**Major Comment 3: Existing studies have already reported on passenger and freight travel patterns in Korea.**

You mentioned that there are several studies addressing passenger and freight travel patterns in Korea, and provided references to those works.

**Response:** Thank you very much for highlighting these important studies. We acknowledge the existence of numerous valuable works related to urban travel patterns and network analysis in Korea. However, the studies you referenced primarily focus on Seoul and its surrounding metropolitan area, analyzing specific aspects of urban mobility within that region. Our study takes a broader, nationwide perspective by addressing the unique interaction between passenger and freight travel patterns across all regions of Korea using a comprehensive dataset from 2019. This allows us to capture inter-regional dynamics and interactions that are not explored in studies limited to a single metropolitan area. By expanding the scope beyond a single city, we believe our research offers a novel contribution to the existing literature, filling an important gap in understanding the spatial structure of both passenger and freight flows at a national level. This broader approach provides valuable insights for policymakers to enhance transportation networks across the entire country.

**Major Comment 4: The recommendations in the paper are not beneficial for decision-making, and the methodology needs to be revised.**

You suggested that the study’s results and recommendations do not provide practical value for policymakers and that the methodology should be revised to improve its usefulness in decision-making.

**Response:** We sincerely appreciate your feedback regarding the practical implications of the study. In response to your valuable suggestion, we have revised the recommendations to better reflect actionable insights for policymakers. Specifically, we have emphasized how the identified travel patterns between regions can inform transportation planning, resource allocation, and infrastructure development in both urban and rural areas. These revisions aim to ensure that the study provides more tangible benefits for decision-makers.

**Conclusion**

We have carefully considered your feedback and made significant revisions to clarify the research question and better align the paper’s content with its stated objectives. These changes aim to improve the clarity and focus of the manuscript, addressing your concerns. We sincerely appreciate your constructive comments, which have been instrumental in enhancing the quality of our work.
