# Supplementary material for: Passenger and freight travel patterns: A cluster analysis based on urban networks
Source: PLoS One. 2025 Mar 11;20(3):e0318084. doi: 10.1371/journal.pone.0318084 (PMC11896054; doi:10.1371/journal.pone.0318084)
Supplement: Attachment — Submitted filename: Reponse_to_Reviewer3_1223.docx [file pone.0318084.s004.docx]

**Dear Reviewer 3,**

Thank you for highlighting the need to provide data from the analysis section to support the arguments made in the discussion. We deeply appreciate your detailed feedback, which has helped us ensure the manuscript is more robust and logically sound. Below, we have addressed each line referenced in your comment, explicitly linking the arguments in the discussion section to the data and figures presented in the analysis section.

**Major Comment 1: “In Discussions Section, 'This travel pattern also aligns with the view presented in Adams [3]', Please provide data from analysis section to support the argument wherever you have written this line in discussion. line 563, 573, 584, 593, 603, 620, 633.”**

**Response:**

(Line 563)
"This travel pattern also aligns with the view presented in Adams [3], which states that such interactions form a broad network."
⇒ This statement highlights the interconnected nature of passenger travel, as identified in our analysis. In the analysis section, **Fig. 3 (Passenger travel patterns)** explicitly demonstrates the dispersed and widespread nature of passenger travel flows across regions. The map shows how passenger travel covers a broader range, connecting multiple cities, including medium-sized cities and surrounding areas. This directly supports Adams' [3] argument, which emphasizes that cities function as interconnected components of a larger system rather than existing in isolation.
In the revised manuscript, we have added the following clarification in the discussion:
"This travel pattern also aligns with the view presented in Adams [3], which states that such interactions form a broad network. As demonstrated in Fig. 3, passenger travel patterns are dispersed and establish extensive regional connectivity, affirming the interconnected system described by Adams."

(Line 573)
"This aligns with Meijer’s [27] the urban network theory, which argues that inter-city cooperation optimizes logistics flows and functions as an efficient system."
⇒ Our freight travel analysis directly supports Meijer’s [27] urban network theory. **Fig. 4 (Freight travel patterns)** and **Fig. 6 (Freight travel network clusters)** show that freight travel is concentrated around specific logistical hubs, such as industrial complexes and ports. These clusters reveal inter-city cooperation to optimize logistics flows. Additionally, the results of cluster formation (Fig. 6) indicate that freight travel forms fewer but geographically broader clusters, which reflects optimized logistics functions across specific economic hubs.
In the revised manuscript, we have updated the discussion to clarify this connection:
"This aligns with Meijer’s [27] urban network theory, which argues that inter-city cooperation optimizes logistics flows and functions as an efficient system. As shown in Fig. 4 and Fig. 6, freight travel clusters are concentrated around specific logistical hubs, reflecting cooperative optimization for economic efficiency."

(Line 584)
"This aligns with the argument presented by Burger and Meijers [10], which suggests that inter-city networks are functionally complementary and can expand economic scale through this cooperation."
⇒ The functional complementarity between passenger and freight travel is evidenced in our analysis. **Fig. 6 (Freight travel network clusters)** demonstrates the spatial distribution of freight clusters, which focus on economic flows, while **Fig. 5 (Passenger travel network clusters)** highlights the shorter, more localized clusters for passenger travel. Together, these figures reveal the complementary roles of passenger and freight travel within the urban network, which aligns with Burger and Meijers’ argument.
In the revised manuscript, the discussion has been expanded to state:
"This aligns with the argument presented by Burger and Meijers [10], which suggests that inter-city networks are functionally complementary and can expand economic scale through cooperation. As highlighted in Fig. 5 and Fig. 6, the distinct clustering patterns of passenger and freight travel illustrate how these networks complement each other in serving diverse urban functions."

(Line 593)
"This is consistent with the findings of Frenken and Hoekman [4], who stated that urban networks focus on connecting specific routes and economic hubs."
⇒ The analysis of freight travel, particularly in **Fig. 4 (Freight travel patterns)** and **Fig. 6 (Freight travel network clusters)**, illustrates that freight travel prioritizes connections between economic hubs such as industrial complexes and ports. These patterns align closely with Frenken and Hoekman’s [4] findings, which emphasize the importance of economic hubs within urban networks.
In the revised manuscript, we have included the following clarification:
"This is consistent with the findings of Frenken and Hoekman [4], who stated that urban networks focus on connecting specific routes and economic hubs. As shown in Fig. 4 and Fig. 6, freight travel prioritizes connections between industrial complexes, ports, and logistics centers, highlighting the centrality of economic hubs in urban networks."

(Line 603)
"This aligns with Batten [1], who stated that personal travel patterns in urban systems are dispersed, as well as Burger and Meijers [10], who suggested that passenger travel forms clusters due to connections to various destinations rather than concentrations along specific routes."
⇒ Our analysis supports these claims through **Fig. 3 (Passenger travel patterns)** and **Fig. 5 (Passenger travel network clusters)**. Passenger travel patterns are highly dispersed, as shown in Fig. 3, covering a broader range of regions. Meanwhile, Fig. 5 highlights the localized clustering of passenger travel, reflecting the connections to diverse destinations, as described by Burger and Meijers [10].
We have revised the discussion as follows:
"This aligns with Batten [1], who stated that personal travel patterns in urban systems are dispersed, as well as Burger and Meijers [10], who suggested that passenger travel forms clusters due to connections to various destinations rather than concentrations along specific routes. As shown in Fig. 3 and Fig. 5, passenger travel patterns are spatially dispersed yet form localized clusters for diverse purposes such as commuting, shopping, and leisure."

(Line 620)
"This is consistent with Capello’s [28] assertion that freight travel follows long-distance economic flows, as well as González [18], who suggested that central areas are formed based on logistical efficiency and economic objectives."
⇒ The long-distance economic flows in freight travel are clearly illustrated in **Fig. 4 (Freight travel patterns)** and **Fig. 8 (Distance between central areas of freight travel)**. These figures demonstrate the spatial distribution of freight flows between distant hubs and the centrality of regions with logistical efficiency, as described by Capello [28] and González [18].
We have revised the discussion to state:
"This is consistent with Capello’s [28] assertion that freight travel follows long-distance economic flows, as well as González [18], who suggested that central areas are formed based on logistical efficiency and economic objectives. As demonstrated in Fig. 4 and Fig. 8, freight travel connects distant hubs based on economic flows, with central areas strategically located to maximize logistical efficiency."

(Line 633)
"These differences in travel patterns demonstrate that the two modes of transport play distinct roles within the urban network. This is consistent with the findings of several studies [24,26,30], which explain how the economic efficiency of urban networks is reflected in travel patterns."
⇒ The distinct roles of passenger and freight travel within urban networks are evidenced by **Fig. 7 (Passenger travel central areas)** and **Fig. 8 (Freight travel central areas)**. These figures highlight the different spatial centralities and functional roles of passenger and freight travel.
The revised manuscript now includes the following statement:
"These differences in travel patterns demonstrate that the two modes of transport play distinct roles within the urban network. As shown in Fig. 7 and Fig. 8, passenger travel reflects short-distance, dense centralities, whereas freight travel demonstrates long-distance connections between economic hubs, supporting the findings of prior studies [24,26,30]."

We hope these detailed revisions address your concerns and strengthen the alignment between the discussion and the analysis. Thank you once again for your valuable feedback, which has greatly improved the clarity and robustness of our manuscript.

**Major Comment 2: “In conclusion section the conclusions must be supported by data from the analysis and discussion sections.”**

**Response:**

Thank you for your valuable feedback and for pointing out the need to ensure that the conclusions are fully supported by data and analysis from the earlier sections of the manuscript. We have carefully revisited the Conclusion section and made significant revisions to align it more closely with the results and discussion presented in the manuscript.

1. **Integration of Key Findings:**
   Each point in the revised Conclusion is now explicitly linked to the data and figures discussed in the Results and Discussion sections. For example:
   - The higher volume of passenger travel and its short-distance nature, highlighted in the Conclusion, is directly supported by the analysis of passenger travel patterns shown in Fig. 3 and discussed on [specific page/line reference].
   - The long-distance and concentrated nature of freight travel, emphasized in the Conclusion, draws on the analysis illustrated in Fig. 4 and elaborated in the Discussion section.
   - The distinction between passenger and freight travel clusters is underpinned by the regular equivalence analysis results presented in Figs. 5 and 6.
2. **Structural and Centrality Insights:**
   The differences in spatial distributions of central areas between passenger and freight travel, as highlighted in the power centrality analysis, are directly tied to Figs. 7 and 8. The Discussion section elaborates on these differences, explaining their implications for regional accessibility and logistical efficiency. These insights have been distilled into the Conclusion, ensuring continuity and support from the data.
3. **Strategic Implications:**
   The strategic need for integrating passenger and freight networks to enhance regional connectivity is not only informed by the figures and analysis but also directly supported by observations discussed throughout the manuscript. The proposed strategies, such as linking multi-nodal passenger activity centers with freight hubs, derive from the observed network characteristics and are grounded in the data presented.
4. **Enhancements to Clarity and Relevance:**
   To ensure that the Conclusion is fully data-driven, we avoided introducing any new concepts or unsubstantiated claims. Each statement in the Conclusion now directly reflects the findings detailed in the Results and Discussion sections, offering a cohesive summary of the study’s contributions.

We believe these revisions address your concerns and demonstrate how the conclusions are fully grounded in the presented data and analysis. Your suggestion has significantly enhanced the clarity and rigor of our manuscript, and we are deeply grateful for your insightful feedback.

Should you have any additional suggestions or require further clarification, we would be more than happy to address them.

Thank you for your time and thoughtful review.
