# Supplementary material for: Passenger and freight travel patterns: A cluster analysis based on urban networks
Source: PLoS One. 2025 Mar 11;20(3):e0318084. doi: 10.1371/journal.pone.0318084 (PMC11896054; doi:10.1371/journal.pone.0318084)
Supplement: Attachment — Submitted filename: Reponse_to_Reviewer3_1124.docx [file pone.0318084.s003.docx]

**Dear Reviewer 4,**

We would like to express our deepest gratitude for your thoughtful and encouraging feedback. Your insightful comments and suggestions have been invaluable in improving the quality and clarity of our manuscript. We are particularly grateful for your recognition of the amendments we made to clarify the figures and enhance the recommendations section. Your detailed guidance allowed us to present our findings in a more professional and coherent manner.

We also appreciate your understanding regarding our use of the 2019 data due to its availability, and we are thankful for your acknowledgement of the distinctions we clarified between our study and other related research. Your feedback on this matter helped us refine the scope and originality of our work.

Furthermore, we are pleased to know that you found the additional amendments based on the opinions of other reviewers to be beneficial. It was indeed our priority to ensure that all comments were carefully considered and integrated into the revised version.

Once again, we sincerely thank you for your kind and supportive review. Your constructive feedback has greatly contributed to strengthening our manuscript, and we are truly grateful for your valuable input throughout this process.

With our highest respect and appreciation.
